# Architects, Hospitals, and Asylums

**Published:** 1887-11-05

**Authors:** Henry C. Burdett

**Affiliations:** Chairman of the Executive Committee of The Hospitals Association and Founder of The Home Hospitals Association


### Architects, Hospitals, and Asylums.

By Henry C. Burdett.
Chairman of the Executive Committee of The Hospitals Association and Founder of The Home Hospitals Association.

The following is the substance of Mr. Burdett's speech in proposing the toast of "The Architects" at the luncheon following the opening ceremony of the latest circular hospital (the Hastings, St. Leonards, and East Sussex), at Hastings :—

Mr. Mayor, Ladies and Gentlemen,—I desire to say a word or two to-day on the subject of architects. I take it that we, who are not architects, but who have to occupy buildings, do not always realise how those buildings are constructed, or what due regard to the proper principles of construction means to each one of us, who have very often to live in them for the greater part of our lives. You know, of course, there are architects and architects. If I were asked, "What is an architect?" I should refer you first of all to the fact that if you went back to the days of the Greeks you would find with them a great architect used to say, "I was inspired to build;" but the Latin architect said boldly, "I built it." Now I take it as there was this marked difference between the Latin and Greek, so there is a difference between architects and architects to-day. There are a class of architects who look upon architecture and their work mainly as a matter of business; there are architects who look upon their work not only as a matter of business, but as a serious responsibility and pleasure. The architect who belongs to the former class, if called in to design and erect a building, would feel it a matter of business to accept the commission, and to proceed at once with every confidence to put up a building on any site presented to him for that purpose. The other architect—and I am proud to think and to know that there are a great number of those gentlemen in the architectural profession in this country to-day—would first of all consider what experience he himself had had in that particular line of work, and if the true principles of construction and the right application of those principles were material factors, and likely to remain so, to those who had to occupy the building which he was asked to undertake; then I make bold to say he would pause, and if he was not acquainted with, or if he had not made a special study of that class of building, he would suggest that another member of the profession should be entrusted with it.

Dr. Bristowe has told you that he, years ago, was called in with Mr. Holmes to make an inspection of all hospitals in Great Britain in Ireland. That report, which appears in the sixth report of the Local Government Board, is a classic in connection with hospital literature, and it is not merely a coincidence, but it is a fact which I am glad to call to mind, that I have been impelled or called upon, as it were, as a younger man to follow up that work, and to extend that work to the medical institutions, not only of this country, but throughout the world. It was in connection with this undertaking that I first of all became acquainted with your architects who have erected this hospital. I had to look round to find some one in the profession who would be able from the study he had given to the subject to render me material assistance in taking plans of existing buildings, and in dealing with plans which came to England from foreign countries, to be reduced to order for the purpose of this book. I was fortunate enough to meet with Mr. Henry Hall, and subsequently with Mr. Keith D. Young, who have given much time and attention to this branch of architecture. They have indeed had an exceptional opportunity of studying closely the plans of hospitals in all parts of the world.

This work, "Hospitals and Asylums of the World," has occupied many years already, but will, I hope, be completed next year. It has given me a wide and intimate, and I believe a unique experience of the architectural profession, which has excited my admiration and compelled my respect. These investigations have necessarily brought me into communication with the managers and officials of the various hospitals and institutions as well as with the architects, and I am able to say that had it not been for the spontaneous kindness and ready co-operation of the architects almost everywhere, the information requisite to complete the book in question could never have been obtained. Let me give an example or two in illustration: The Foreign and the Colonial Offices have done their best to afford me every assistance in collecting information and plans. In some countries it has been found difficult or impossible for the Government to procure the information desired, and in these cases direct communication had to be addressed to the managers of the institutions, failing whom the architects have been applied to. Now, it is a fact, and one honourable alike to a great profession and worthy of public recognition, that Governments might be powerless and managers of institutions indifferent, but the architects, almost without exception, have been found courteous, helpful, and ready co-operators in this work, which has been undertaken in what is believed to be the interest of hospital construction and administration, using the word hospital in its widest and most extended sense. I feel myself, therefore, to be under a lasting debt to the architectural profession, and I desire it to be known as widely as possible that the hundreds, nay, thousands of plans which have been collected will always be ready for the inspection of any member of that profession who may take an interest in this class of buildings, their planning, arrangement, and completeness. I desire to take the present opportunity of thanking this great army—for it is a great army—of unknown but greatly-appreciated co-operators in a work of no little importance and interest. My own indebtedness to them I shall never be able to discharge, but I could wish that I might make my voice heard and my own gratitude known to every architect in the world who has devoted time and money to the preparation of plans to assist me in the work to which I have here alluded. I do not wish to be misunderstood in any way, but it has been my privilege or misfortune to be called in as assessor or arbitrator in cases of competition drawings, where a new hospital had to be erected. These competitions, and the methods by which a selection is often made from a number of plans, seem to me to need revision in justice to the architects themselves and in order to secure the interest of all concerned. I have said that there are architects who would exercise self-denial sufficient to refuse to prepare plans for a new hospital or a new asylum if they felt that they had had no special experience in the work, and that there were other members of the profession who had devoted themselves to this branch with success. Now specialism has its drawbacks and abuses, but I do feel, and I hope that you agree with me, that the housing of the sick, and indeed the housing of any number of human beings in a public institution, is a matter of very great importance and responsibility, and that the self-denial I have referred to is not only wise but judicious, and to be commended and encouraged to the utmost.

Under existing conditions this principle of self-denial, that is, the wise utilisation of special knowledge on the part of the members of the architectural profession and of others, is not sufficiently borne in mind when an assessor or arbitrator is to be appointed to make a selection from a number of drawings and plans sent in for competition. I venture to think and hope that the action recently taken by the Royal Institute of British Architects, and by the architectural profession in America, will lead to a reconsideration of this question, and to an arrangement whereby such competitions will be decided in the near future, not by one but by two assessors, one of whom represents the architects and the other a trained and experienced expert possessed of thorough knowledge of the management and requirements of the hospital or asylum to be erected, and of the hygienic conditions which ought to be observed in the construction of such a building. This principle has already been successfully adopted by individual architects when preparing drawings for competition and for clients, and I have been pleased to find an important improvement in the class and character of the plans submitted in recent years. A capable architect, Mr. Jacobs, for instance, has, I believe, always associated with himself one of the ablest and most experienced asylum superintendents when he has had this class of work in hand. Why should not this principle be generally adopted, both by individual architects and by those public bodies and others who put up new buildings to competition? It would obviate much heartburning and dissatisfaction, and would prevent the erection of not a few incomplete and unsatisfactory buildings, because under such conditions the plan selected would almost certainly be not only the best architecturally, but also the best structurally and hygienically, and from the point of view of the practical administrator.

There is one other point about architects which is worthy of consideration. You have no doubt observed to-day the excellence of the building as such; that is to say, the materials and the workmanship must commend themselves more and more to the visitor in proportion to the technical knowledge he has of what constitutes good work. Of course there are builders and builders, as there are architects and architects; but I take it that the builder of this hospital, who is present to-day, will agree with me that the architect can, and this materially, influence the character and quality of the work put into the buildings which he may design. If his specifications are carefully prepared and exhaustive—and these can neither be carefully prepared nor exhaustive unless the architect has a sound practical knowledge of the details which collectively make up and secure efficiency in the administration of large public buildings—then extras will not be necessary, because the whole work as such has been thought out from first to last, and in these circumstances forgetfulness and oversight are words not to be found in the vocabulary of the architect. Therefore I say, and I say it with a feeling of considerable responsibility, that this question of extras—that is, the allowances over and above the contracts which are placed before those who have to pay for the building—may be taken as an index to the capacity and the care of the architect in a way that very little else can. I do not of course mean the extras which are the result of the constant changes introduced at the instigation of the employer as the building progresses, because I take it that if the public who employ architects presume to judge them, such judges will feel themselves bound in honour to exercise equal care and forethought to that which, when exhibited by the architect, reduces extras to a minimum. Indeed, in the case—and it is a memorable case—of the first circular hospitol erected in England, namely, the Miller Memorial Hospital at Greenwich, so thoroughly did the architects do their work that there were not only no extras, but there was positively something to spare when the accounts were finally adjusted. The *British Medical Journal* wisely remarked at the time that such a fact as this ought to be written up in letters of gold in the hospital board-room, so that all who came to see the building might take the fact to heart, and say whether they and their architects could not go and do likewise.

Now this brings me to the immediate subject of the toast which I have to propose, namely, that of Messrs. Young and Hall, who built the Miller Memorial Hospital, and who have this day handed over to you another hospital, which I make bold to say is by far the completest and most perfect building on the circular principle which has yet been erected in this country or abroad. I believe it was stated to-day that Burnley Hospital was the first complete hospital ever built in England on the circular system. If that was said it is an error. Before the Burnley Hospital plans were accepted Greenwich Hospital had been built, and I myself was consulted before the final selection was made at Burnley, with the view of ascertaining whether or not the circular principle was a sound principle, and one which the medical staff would be able to recommend with confidence. As a matter of fact, the assessor had selected a plan on the rectangular principle, but a member of the medical staff, the senior surgeon, Mr. Brown, hearing about Greenwich, and being familiar with the writings of Professor Marshall and Mr. Gordon Smith, determined he would have this question reconsidered. In the result the committee came to Greenwich, inspected the circular wards there, and Burnley Hospital was erected, not from the rectangular plans, but from new plans on the circular principle. I mention this fact because history is often apt to get mixed, and as you open to-day what I regard as the most perfect type of circular hospital in this country, I wish you to clearly understand where the work commenced, and where you stand in connection with that work. I say that yours is the most perfect and the most complete specimen of the circular form of hospital erection, for the reason that you have avoided the dangers and the errors of the circular principle, and you have been content to have your wards of proper dimensions, and to devote the central portions of those wards to the flues for ventilation and the fireplaces. Now I venture to say, that if any of you are in the North, and will take the trouble to go to Burnley, you will recognise at once, whether you are acquainted with the principles of hospital construction or not, that I am speaking quite to book, and am accurately defining the position which your hospital ought to occupy as a building constructed on the circular principle.

The Governors, the Committee, and the Medical Staff are to be congratulated on having selected this type of building, and administratively you will find it will prove a great economy in the expenditure of labour and of individual exertion, and so give increased comfort and satisfaction to the sufferers who have to find there a temporary shelter in the hour of illness. I say that, because I know, as one who has had charge of rectangular hospitals for many years, that a large rectangular ward, where the nurse's room is at one end, and it may be the worst case—*i.e.*, the patient who is most severely ill—at the other, that the nurse, however devoted she may be, as the day goes on must find the whole length of that ward a great strain upon her physically. If she is not zealous, it must be a great temptation to her not to go quite so readily, and possibly not quite so often, to look after that specially urgent case such a long distance away. The effect of a circular ward is this, that the nurse can see all the beds except one, certainly except two, in the whole ward, from any point in it; and she has to travel the shortest possible distance to get to any one patient who may need her services at a given time. In saying this I wish to echo and confirm the views expressed by Dr. Bristowe, that we must not yet regard the circular ward as the most perfect system of hospital construction. We must but look upon it as one type of construction suited to special cases, and one which deserves a fair and prolonged trial. It is simply as one who desires to see this new principle fairly and honestly and squarely tried, that I rejoice to think, having regard especially to your site, that you have put up such excellent circular wards as you have done.

Now one word as to your architects, and I have done. I do not know whether it strikes you, but it struck me to-day, going over your building, that it is eloquent with testimony to the fact that those who designed that building are familiar with the requirements, the administrative requirements of a hospital, and that in every particular you will find due regard has been paid to economy of labour, and consequently to efficiency. That point has struck me very much. It is, of course, if I may say so, a matter that I should criticise rather severely, coming down, as I have done at your invitation, to inspect this hospital, because I know Mr. Young and Mr. Hall ought to be able to provide you with a proper and complete hospital. I am therefore gratified to find the opportunities they have had of making themselves familiar with the newest and best hospital appliances and methods have been made the most of, and that I may honestly and sincerely congratulate this town upon having built such an excellent hospital in this year of Jubilee. I believe you will find you will have many visitors, not only Englishmen, but from foreign countries too, who will come to see the Hastings Hospital, because having seen the Antwerp Hospital, the hospital at Hampstead, the Circular Hospitals for the Army, and the hospitals at Burnley and Greenwich, I am able to say you have here to-day the most typical, and I believe the most complete building on the circular principle which has yet been erected either in this country or indeed anywhere.

